# Diaminoterephthalate–α-lipoic acid conjugates with fluorinated residues

**DOI:** 10.3762/bjoc.15.96

**Published:** 2019-04-26

**Authors:** Leon Buschbeck, Aleksandra Markovic, Gunther Wittstock, Jens Christoffers

**Affiliations:** 1 Institut für Chemie, Universität Oldenburg, Carl von Ossietzky-Str. 9–11, D-26129 Oldenburg, Germany

**Keywords:** chromophore, diaminoterephthalate, fluorine surface marker, fluorescence dye, lipoic acid, self-assembled monolayers

## Abstract

Two bifunctional diaminoterephthalate (DAT) fluorescence dyes were prepared in a three-step sequence including one deprotection reaction. One functional unit is α-lipoic acid (ALA) for binding the dye to gold surfaces. It was introduced to the DAT scaffold by an amidation reaction. The other functional unit is a *para*-(trifluoromethyl)benzyl group for facile detection of the surface-bound material by X-ray photoelectron spectroscopy (XPS). This residue was introduced by reductive amination of the DAT scaffold with the respective benzaldehyde derivative. In one compound (60% yield over three steps) the ALA unit is directly bound to the DAT as a relatively electron-withdrawing amide. In solution (CH_2_Cl_2_), this material shows strong fluorescence (quantum yield 57% with emission at 495 nm, absorption maximum at 420 nm). The other compound (57% yield over three steps) possesses a propylene spacer between the ALA and the DAT units for electronic decoupling, thus, bathochromic shifts are observed (absorption at 514 nm, emission at 566 nm). The quantum yield is, however, lower (4%). Self-assembled monolayers on a gold surface of both compounds were prepared and characterized by high-resolution XPS of the C 1s, O 1s, S 2p, N 1s and F 1s emissions. The high signal-to-noise ratios of the F 1s peaks indicated that trifluoromethylation is an excellent tool for the detection of surface-bound materials by XPS.

## Introduction

**Figure 1:**
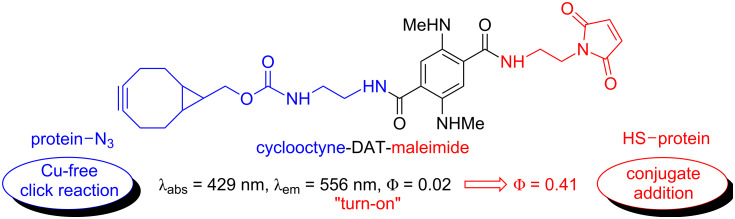
Bifunctional DAT as a cross-linker for proteins. The compound is a turn-on-probe, i.e., the fluorescence quantum yield Φ = 2% increased upon reaction with the target to Φ = 41%.

Diaminoterephthalates (DATs) are powerful fluorescence dyes [1–2] with outstanding properties such as high quantum yields and pronounced stability against photobleaching [3–5]. Although being structurally relatively simple, this class of dyes is so far underrated in the literature. The chromophore, which is accessed from succinyl succinates and primary amines [6–7], can be regarded as a molecular scaffold [8], which can be orthogonally equipped with different functional units [9] by simple transformations. Thus, different applications in materials science [10] and life sciences [11–12] can be addressed by tailored functional DATs. As an example for an application in biochemistry, Figure 1 shows a compound with cyclooctyne and maleimide as functional units. It was used as a "turn-on" fluorescence probe for cross-linking proteins [13]. The highly reactive cyclooctyne residue undergoes 1,3-dipolar cycloadditions with organoazides (copper-free click reactions) [14]. The second functional unit, the maleimide moiety, is a reactive probe for mercaptane, which could be, e.g., a protein holding a cysteine residue on its surface [15–17]. The successful ligation by conjugated addition can be followed by the changes of the fluorescence quantum yields (i.e., "turn-on effect") [11–13].

Modification of solid surfaces by defined layers of organic compounds raised significant interest in the last century. Those modifications can be fairly simple as in the case of alkanethiolate layers on gold [18] yet have a number of applications in sensorics [19], microcontact printing [20], dip-pen nanolithography [21], microfluidics [22], and protection of nanoparticles [23]. Initially, surface modification aimed on controlling physical properties of surfaces [24–25], while nowadays chemical surface properties can be tuned to yield platforms for the study of electron transfer [26–27] or for building surface molecular devices for different purposes, commonly called integrated molecular systems [19]. These molecular systems are mainly used for pH sensing [28–30], inorganic- [31–33], organic- and biosensors [34–35]. Self-assembled monolayers (SAMs) can also be triggered electrochemically to perform reactions on the surfaces. These "dynamic" surfaces allow the "turn-on" of active states upon application of electrochemical potentials, for instance, for the addition of compounds to surfaces or for the control of cell adhesion [36–38].

Characterization of such integrated molecular systems constitutes a substantial challenge (as compared to the structural characterization of soluble organic compounds) because (1) different compounds are potentially present on the surface and cannot be separated; (2) some powerful techniques, especially NMR and MS with soft ionizations are not applicable; and (3) the total amount of material is extremely small (i.e., about 10^−9^ mol·cm^−2^) [39]. X-ray photoelectron spectroscopy (XPS) is one of the few suitable methods for detection of surface-immobilized compounds and changes on the surfaces. However, due to similar chemical shifts in binding energies, fragments of larger organic compounds are not easily distinguished. Labeling molecular entities with elements possessing large excitation cross sections like fluorine, chlorine, or bromine [40] represents an approach that can greatly simplify the detection of molecular reactions (cleavage, anchoring) in monolayers after their assembly on surfaces.

In the course of our project on surface modifications by redox-active SAMs, we envisioned a bifunctionalized DAT as suitable building block. Surface binding to a metal support, e.g., gold, should be accomplished by an α-lipoic acid (ALA) residue. Furthermore, a fluorine-substituted moiety bound to DAT shall facilitate detection by XPS.

## Results and Discussion

**Scheme 1:**
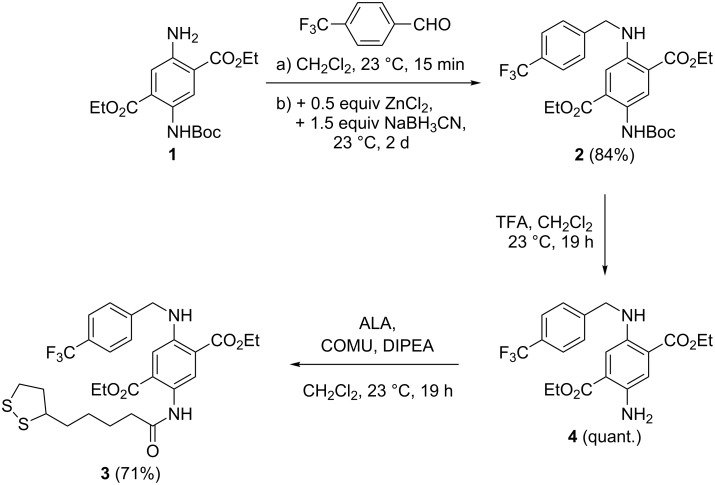
Preparation of DAT–ALA conjugate **3** with fluorinated benzyl residue.

**Synthesis.** The preparation of DAT–ALA conjugate **3** with a fluorinated residue started from mono-carbamate-protected diethyl DAT **1** (Scheme 1). Compound **1** was accessed in three steps from diethyl succinate according to Wu et al. [41]. Reductive amination with trifluoromethylated benzaldehyde was accomplished with a mixture of ZnCl_2_ and NaBH_3_CN [42] yielding the respective *N*-benzylated compound **2** in good yield. After subsequent *N*-Boc-deprotection with TFA (product **4** in quantitative yield), the primary amino function was amidated with racemic α-lipoic acid (ALA) in the presence of COMU–DIPEA [COMU = (1-cyano-2-ethoxy-2-oxoethylideneaminooxy)(dimethylamino)(morpholino)carbenium hexafluorophosphate, DIPEA = ethyldiisopropylamine] [43] as coupling reagent to the first title compound **3** in 71% yield. Due to the electron-withdrawing amide group, the chromophore is relatively electron deficient (absorption at 420 nm and emission at 495 nm).

**Scheme 2:**
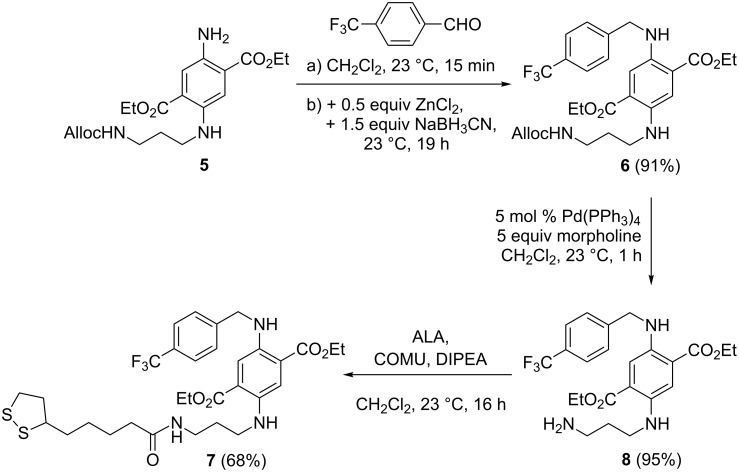
Preparation of conjugate **7** consisting of fluorinated DAT and ALA moieties with an additional propylene linker unit.

In order to achieve a bathochromic shift of absorption and emission bands, the DAT and ALA moieties should be electronically decoupled by introduction of a propylene spacer. Therefore, we started the synthesis with compound **5** (Scheme 2), which was accessed from compound **1** in two steps by reductive amination with *N*-Alloc-3-aminopropanal and subsequent *N*-Boc deprotection as reported recently [44]. Reductive amination with trifluoromethylated benzaldehyde was accomplished as described for compound **2** and furnished product **6** in 91% yield. The Alloc-protecting group was then cleaved (95% yield of product **8**) in a palladium-catalyzed allylic substitution reaction with morpholine as a scavenger of the allylic cation [45–46]. Finally, the primary amine **8** was coupled with ALA in the presence of COMU–DIPEA to furnish the second title compound **7** in 68% yield. Indeed, a bathochromic shift of the spectral data was observed (absorption at 514 nm and emission at 566 nm).

**Spectroscopy.** Being typical push–pull aromatic systems, all DAT derivatives are colored materials (yellow, orange, or red) showing pronounced fluorescence in solution (Table 1). The absorption and emission wavelengths are sensitively tuned by the electron-accepting or electron-donating nature of the nitrogen-substituents at the aromatic ring. In Table 1, compound **1** with an NH_2_ and NHBoc group is the most electron-deficient one with absorption and emission wavelength at 408 nm and 487 nm, respectively (Stokes shift ca. 80 nm). A bathochromic shift of 16 nm of both, absorption and emission wavelengths, is achieved by introduction of a benzyl residue at one nitrogen atom (compound **2**). If the Boc group is replaced by the slightly more electron-withdrawing carboxamide as in compound **3**, this bathochromic shift (compared to compound **1**) is only ca. 10 nm. Without an *N*-acceptor moiety, i.e., *N*-monoalkyl (compounds **4** and **5**) or *N*,*N'*-dialkyl substitution (compounds **6**–**8**), a stronger bathochromic shift is observed towards the absorption at 451–473 nm (514 nm for compound **7**, which is exceptionally high) and emission at 547–567 nm. The quantum yields of compounds **1**–**4** range between 0.21 and 0.57, for compounds **5**–**8** with propylene linker, the quantum yields are between 0.04–0.13. Interestingly, target compound **3** has the highest (0.57), target compound **7** the lowest (0.04) quantum yield.

**Table 1:** Spectroscopic properties of diaminoterephthalates **1**–**8**; solvent CH_2_Cl_2_.

Compound	λ_max_ [nm]	lg ε [dm^3^·mol^−1^·cm^−1^]	λ_em_ [nm]^a^	Φ^b^

**1**	408	3.87	487	0.21
**2**	424	3.88	503	0.36
**3**	420	3.67	495	0.57
**4**	451	3.72	547	0.21
**5**	455	3.77	553	0.13
**6**	473	3.83	567	0.12
**7**	514	3.58	566	0.04
**8**^c^	464	3.58	566	0.09

^a^Excitation at λ_max_ of the absorption band. ^b^Quantum yields were determined according to the Parker Rees method [47–49] using rhodamine B in EtOH as standard [λ_max_ = 544 nm, lg ε [dm^3^·mol^−1^·cm^−1^] = 3.23, λ_em_ = 569 nm, Φ = 0.46] [50–51]. ^c^In MeOH as solvent.

**Figure 2:**
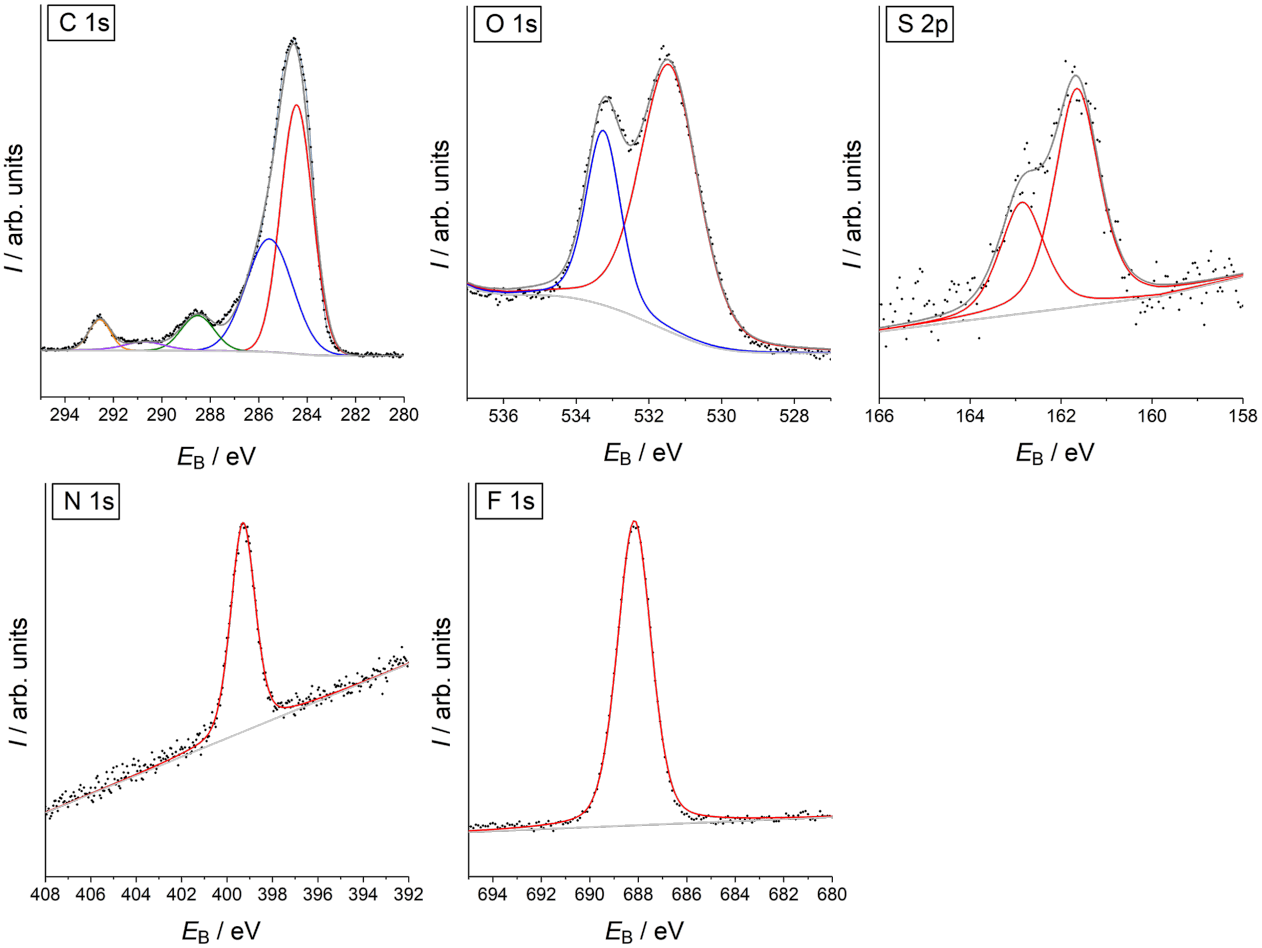
High-resolution XPS of SAM **3**.

**Figure 3:**
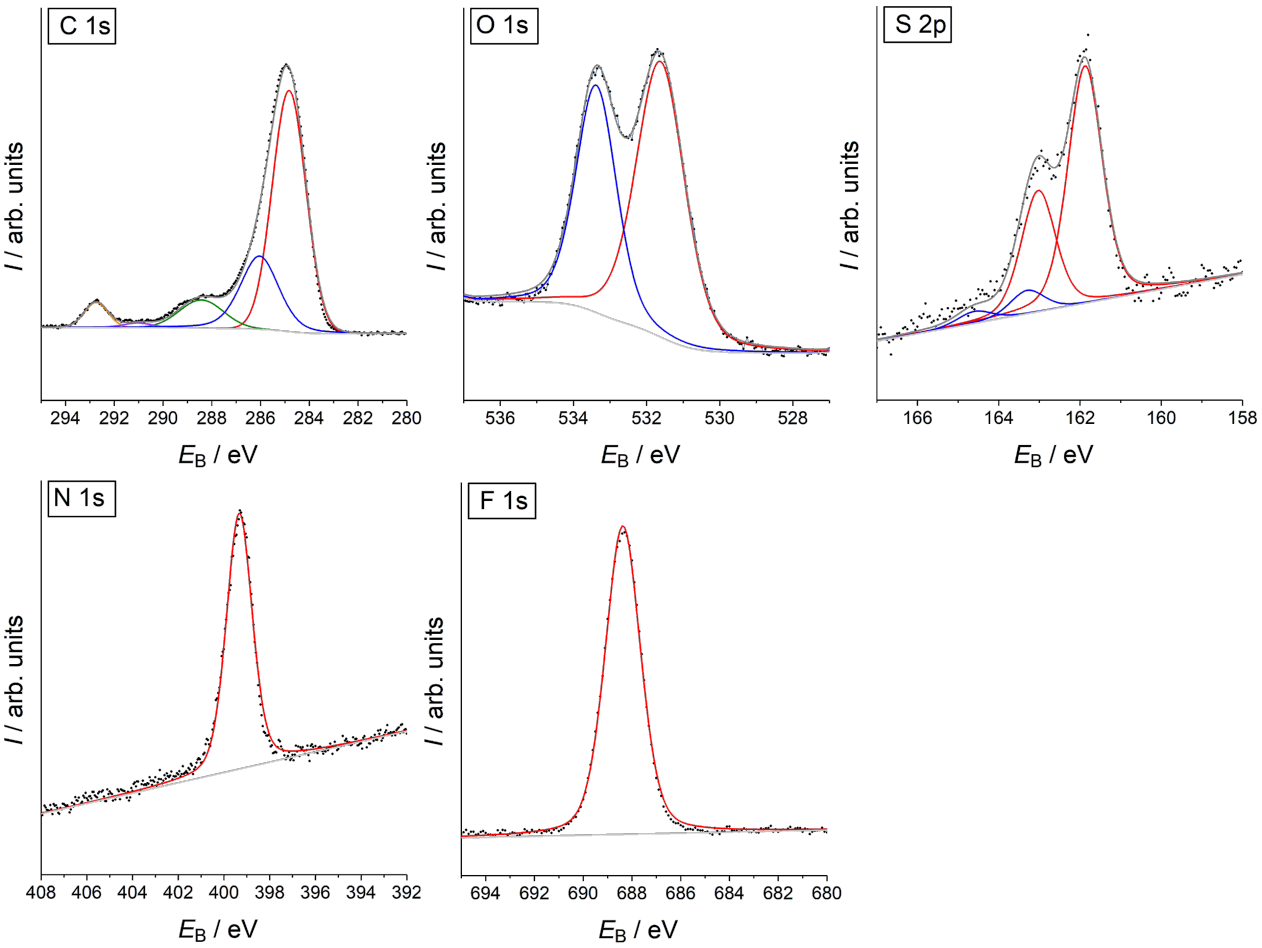
High-resolution XPS of SAM **7**.

**XPS characterization of SAMs.** SAMs were prepared from compounds **3** and **7** exploiting the strong binding affinity of the ALA residue to gold surfaces [52]. The resulting layers of compound **3** (SAM **3**) and **7** (SAM **7**) were characterized by XPS of the C 1s, O 1s, S 2p, N 1s and F 1s emissions (Figure 2 for SAM **3**, Figure 3 for SAM **7**).

The largest contributions of the C 1s spectra at 284.4 eV (SAM **3**) and 284.7 eV (SAM **7**) correspond to the carbon atoms of the alkyl chains, the aromatic ring and the carbon bound to sulfur atoms (Table 2). The peak at 285.5 eV (SAM **3** and SAM **7**) corresponds to the carbon atoms with a higher binding energy due to the bond to one more electronegative atom (*C*−O and *C*−N) [53]. Figure 2 and Figure 3 show further C 1s peaks lying at binding energies of 288.5 eV, 290.6 eV, and 292.6 eV (SAM **3**) and 288.5 eV, 290.9 eV, and 292.7 eV (SAM **7**). The first component at 288.5 eV corresponds to the carbonyl carbon atom in the amide and ester functions, which are bond to two electronegative elements (O=*C*−O and O=*C*−N) that shift the binding energies to higher values. The broad contributions at 290.6 eV (SAM **3**) and 290.9 eV (SAM **7**) originate from multi-electron excitations, i.e., from shake-up process at aromatic systems [54]. The components at 292.6 eV and 292.7 eV correspond to the carbon bound to three fluorine atoms causing a strong shift of binding energies outside the range typical for most organic compounds.

**Table 2:** Binding energies of the C 1s, O 1s, S 2p, N 1s, and F 1s photoemission lines of SAM **3** and SAM **7**.

	SAM **3**	SAM **7**		
XPS line	*E*_B_ [eV]	*E*_B_ [eV]	Assignment	References

C 1s	284.4	284.7	*C*–C, *C*–S	[53]
	285.5	285.5	*C*–O, *C*–N	[53]
	288.5	288.5	O=*C*–O, O=*C*–N	[53]
	290.6	290.9	multi electron excitations	[54]
	292.6	292.7	*C*F_3_	[55]
O 1s	531.5	531.6	*O*=C	[56]
	533.2	533.3	*O*–C and residual water	[56–58]
S 2p_3/2_	161.8	161.8	R–*S*–Au	[59]
	–	163.3	radiation damage	[60–62]
S 2p_1/2_	162.9	163.0	R–*S*–Au	[59]
	–	164.6	radiation damage	[60–62]
N 1s	399.3	399.3	*N*–aryl	[63]
F 1s	688.2	688.4	C*F*_3_	[55]

The O 1s spectrum of the monolayers consist of two components at 531.5 eV and 533.2 eV (SAM **3**) and 531.6 eV and 533.3 eV (SAM **7**) for oxygen atoms involved in the *O*=C and *O*−C bonds, respectively [55]. The slight deviation from the peak area ratio of 3:2 for I(531.5 eV)/I(533.5 eV), which is expected from the molecular structure of compounds **3** and **7**, may be caused by residual adsorbed water that also causes an O 1s component at 533.0–533.5 eV [57–58].

The main doublet in the S 2p spectra of both monolayers at 161.8 eV corresponds to sulfur bound to gold [59]. Although, S 2p spectra were recorded first among all spectra there is indication of radiation damage by the doublet at 163.3 eV in SAM **7** (Figure 3) [60–62]. This signal is not found in monolayers from SAM **3** (Figure 2).

There is only one N 1s component at 399.3 eV which corresponds to all nitrogen atoms at both SAMs [63]. Fluorine atoms in the F_3_C group are detected at binding energies of 688.2 eV (SAM **3**) and 688.4 eV (SAM **7**) in agreement with the literature [55], which is a typical value for the F_3_C group. The high signal-to-noise ratio, especially in comparison to the S 2p and N 1s signals, illustrates its suitability for molecular surface labeling.

## Conclusion

Two bifunctional diaminoterephthalate (DAT) fluorescence dyes have been prepared. One functional unit is α-lipoic acid (ALA) for binding the dye to gold surfaces. The other carries a trifluoromethyl group for facile detection of the surface-bound material by X-ray photoelectron spectroscopy (XPS). In the first of two target structures, compound **3**, the fluorinated residue was introduced by reductive amination of mono-*N*-Boc-protected diethyl DAT **1** with *para*-(trifluoromethyl)-substituted benzaldehyde. After *N*-deprotection, the ALA unit was introduced by amidation using a standard coupling protocol (with COMU–DIPEA). Compound **3** was accessed in 60% yield over three steps. In solution (CH_2_Cl_2_), **3** shows strong fluorescence (quantum yield Φ = 57%) at λ_em_ = 495 nm when irradiated at its absorption maximum at λ_max_ = 420 nm. In order to achieve bathochromic shifts of the wavelengths, the electron-withdrawing ALA-amide unit was electronically decoupled from the DAT chromophore by introducing a propylene spacer. Thus, diethyl DAT was first equipped with an *N*-Alloc-protected 3-aminopropyl side chain at one nitrogen function following a literature protocol. This compound, **5**, was then submitted to reductive amination with *para*-(trifluoromethyl)benzaldehyde. After Alloc deprotection, the side chain was amidated with ALA following the same COMU–DIPEA protocol as described for **3**. The second target compound **7** was obtained in 59% yield over three steps. Indeed, the emission and absorption wavelengths in solution (CH_2_Cl_2_) of compound **7** were bathochromically shifted (λ_max_ = 514 nm, λ_em_ = 566 nm). However, the quantum yield of **7** was on the other hand significantly lower (Φ = 4%). Self-assembled monolayers (SAMs) of both compound **3** and **7** were prepared on a gold surface. The elemental compositions of these two SAMs were characterized by high-resolution XPS of the C 1s, O 1s, S 2p, N 1s and F 1s emissions. As is indicated by the high signal-to-noise ratio of the F 1s peaks, the trifluoromethylation is an excellent tool for the detection of surface-bound materials by XPS.

## Experimental

**General experimental methods:** Preparative column chromatography was carried out using Merck SiO_2_ (35–70 µm, type 60 A) with hexanes (mixture of isomers, bp. 64–71 °C), CH_2_Cl_2_, EtOAc and MeOH as eluents. TLC was performed on aluminum plates coated with SiO_2_ F_254_. ^1^H and ^13^C NMR spectra were recorded on a Bruker Avance DRX 500 instrument. Multiplicities of carbon signals were determined with DEPT experiments. HRMS spectra of products were obtained with Waters Q-TOF Premier (ESI) or Thermo Scientific DFS (EI) spectrometers. IR spectra were recorded on a Bruker Tensor 27 spectrometer equipped with a diamond ATR unit. UV–vis spectra were recorded with a Shimadzu UV-1800, fluorescence spectra with a Shimadzu RF-5301PC spectrometer. Compounds **1** [41] and **5** [44] were prepared according to literature procedures. All other starting materials were commercially available.

XPS of SAMs were recorded with ESCALAB 250 iX (Thermo Fisher, East Grinsted, UK) using a monochromatized Al K_α_ excitation (1486.6 eV) and the magnetic lens mode. Data acquisition and spectra processing was performed with the Avantage software v. 5.52. After recording a survey spectrum, high-resolution spectra were obtained from the O 1s, F 1s, N 1s, C 1s and S 2p region with a pass energy of 30 eV, a step size of 0.05 eV and 50 ms dwell time. In order to avoid radiation damage of the SAMs, which becomes evident by the appearance of a second S 2p doublet at 163.3 eV/164.5 eV [60–62], 35 scans were averaged from different regions of the same sample (area scan). The peak-fit analysis used the "smart background" from Avantage software and sum of Gaussian and Lorentzian contributions for each spectral component. The area ratio between S 2p_3/2_ and S 2p_1/2_ emissions from each state was fixed to 2:1. The graphs show the experimental points as dot, the sum curve as black line, the background as grey line and the spectral components as colored lines.

**Diethyl 2-(*tert*-butoxycarbonyl)amino-5-[4-(trifluoromethyl)benzylamino]terephthalate (2):** In a similar manner as described in [44], a solution of DAT **1** (0.567 mmol, 200 mg, 1.0 equiv) in CH_2_Cl_2_ (3 mL) was added dropwise to a cooled (ice-water bath) suspension of 4-(trifluoromethyl)benzaldehyde (0.850 mmol, 148 mg, 1.5 equiv) in CH_2_Cl_2_ (3 mL). After stirring the mixture for 15 min at ambient temperature, it was cooled (ice-water bath) and ZnCl_2_ (0.23 mmol, 39 mg, 0.5 equiv) and NaBH_3_CN (0.85 mmol, 53 mg, 1.5 equiv) were added. Subsequently, the mixture was stirred for 2 d at ambient temperature, then diluted with water (25 mL) and extracted with CH_2_Cl_2_ (3 × 25 mL). The combined organic layers were dried (MgSO_4_) and evaporated after filtration. The residue was recrystallized (from hexanes/EtOAc 8:1, 20 mL) to furnish the title compound **2** (244 mg, 0.478 mmol, 84%) as an orange solid. Mp 210 °C. *R*_f_ = 0.31 (SiO_2_, hexanes/CH_2_Cl_2_ 1:3); ^1^H NMR (500 MHz, CDCl_3_) δ 1.30 (t, *J* = 7.1 Hz, 3H), 1.42 (t, *J* = 7.1 Hz, 3H), 1.51 (s, 9H), 4.28 (q, *J* = 7.1 Hz, 2H), 4.38 (q, *J* = 7.1 Hz, 2H), 4.50 (d, *J* = 5.6 Hz, 2H), 7.16 (s, 1H), 7.48 (d, *J* = 8.0 Hz, 2H), 7.59 (d, *J* = 8.1 Hz, 2H), 7.91 (t, *J* = 5.6 Hz, 1H), 8.86 (s, 1H), 9.49 (br. s, 1H) ppm; ^13^C{^1^H} NMR (125 MHz, CDCl_3_) δ 14.12 (CH_3_), 14.48 (CH_3_), 28.53 (3 CH_3_), 47.19 (CH_2_), 61.28 (CH_2_), 61.63 (CH_2_), 80.23 (C), 113.61 (CH), 115.86 (C), 121.08 (C), 122.82 (C), 124.28 (q, *J* = 272.0 Hz, C), 125.76 (q, *J* = 3.6 Hz, 2 CH), 127.53 (2 CH), 129.67 (q, *J* = 32.3 Hz, C), 130.31 (C), 143.34 (C), 144.74 (C), 153.25 (C), 167.29 (C), 168.17 (C) ppm; IR (ATR): 3310 (w), 2983 (w), 1715 (m), 1676 (m), 1620 (w), 1542 (m), 1469 (w), 1422 (m), 1393 (w), 1368 (w), 1326 (m), 1241 (m), 1217 (s), 1159 (s), 1107 (vs), 1066 (m), 1043 (w), 1022 (s), 907 (w), 870 (w), 830 (m), 786 (m), 764 (w), 700 (w), 654 (w), 610 (w), 596 (w), 575 (w) cm^−1^; MS (EI, 70 eV) *m*/*z* (%): 510 (17) [M^+^], 453 (54), 435 (17), 410 (100), 388 (16), 362 (12), 251 (21), 158 (20); HRMS (EI): [M^+^] calcd for C_25_H_29_F_3_N_2_O_6_^+^, 510.1972; found, 510.1972; UV–vis (CH_2_Cl_2_): λ_max_ (lg ε) = 424 nm (3.88); fluorescence (CH_2_Cl_2_): λ_em_ = 503 nm, λ_ex_ = 424 nm, Φ = 0.36; C_25_H_29_F_3_N_2_O_6_ (510.51 g·mol^−1^).

**Diethyl 2-amino-5-[4-(trifluoromethyl)benzylamino]terephthalate (4):** In a similar manner as described in [44], TFA (3 mL) was added dropwise to a cooled (ice-water bath) solution of carbamate **2** (0.402 mmol, 205 mg) in CH_2_Cl_2_ (3 mL). The mixture was stirred for 19 h at ambient temperature and then poured into saturated aqueous NaHCO_3_ solution (50 mL). After stirring for 5 min at ambient temperature, it was extracted with CH_2_Cl_2_ (3 × 50 mL). The combined organic layers were dried (MgSO_4_) and evaporated after filtration. Chromatography of the residue (SiO_2_, hexanes/EtOAc 6:1 with 1 vol % NEt_3_, *R*_f_ = 0.31) furnished the title compound **4** (171 mg, 0.417 mmol, quant.) as an orange-red solid. Mp 98–99 °C; ^1^H NMR (500 MHz, CDCl_3_) δ 1.30 (t, *J* = 7.1 Hz, 3H), 1.39 (t, *J* = 7.1 Hz, 3H), 4.27 (q, *J* = 7.1 Hz, 2H), 4.34 (q, *J* = 7.1 Hz, 2H), 4.46 (s, 2H), 7.12 (s, 1H), 7.38 (s, 1H), 7.50 (d, *J* = 8.0 Hz, 2H), 7.59 (d, *J* = 8.1 Hz, 2H) ppm, signals for three NH protons were not observed; ^13^C{^1^H} NMR (125 MHz, CDCl_3_) δ 14.28 (CH_3_), 14.42 (CH_3_), 47.73 (CH_2_), 60.93 (CH_2_), 61.93 (CH_2_), 113.88 (CH), 117.33 (C), 117.83 (C), 119.99 (CH), 124.35 (q, *J* = 271.9 Hz, C), 125.68 (q, *J* = 3.6 Hz, 2 CH), 127.64 (2 CH), 129.53 (q, *J* = 32.5 Hz, C), 139.79 (C), 141.69 (C), 143.85 (C), 167.44 (C), 167.82 (C) ppm; IR (ATR): 3421 (w), 2988 (w), 2904 (w), 1679 (s), 1619 (w), 1563 (w), 1514 (m), 1474 (w), 1441 (w), 1416 (w), 1367 (w), 1329 (m), 1289 (m), 1280 (w), 1265 (w), 1207 (vs), 1158 (m), 1102 (vs), 1062 (s), 1018 (m), 878 (w), 844 (w), 827 (w), 787 (m), 729 (w), 655 (w), 596 (w) cm^−1^; MS (EI, 70 eV) *m/z* (%): 410 (100) [M^+^], 362 (16), 251 (42), 204 (22), 158 (52), 132 (18); HRMS (EI): [M^+^] calcd for C_20_H_21_F_3_N_2_O_4_^+^, 410.1448; found, 410.1447; UV–vis (CH_2_Cl_2_): λ_max_ (lg ε) = 451 nm (3.72); fluorescence (CH_2_Cl_2_): λ_em_ = 547 nm, λ_ex_ = 451 nm, Φ = 0.21; C_20_H_21_F_3_N_2_O_4_ (410.39 g·mol^−1^).

***rac*-Diethyl 2-[5-(1,2-dithiolan-3-yl)pentanoylamino]-5-[4-(trifluoromethyl)benzylamino]terephthalate (3):** In a similar manner as described in [44], DIPEA (1.05 mmol, 136 mg, 3.0 equiv) and COMU (1.05 mmol, 450 mg, 3.0 equiv) were added successively to a cooled (ice-water bath) solution of *rac*-α-lipoic acid (0.698 mmol, 144 mg, 2.0 equiv) in CH_2_Cl_2_ (8 mL). After stirring for 1 h at ambient temperature a solution of amine **4** (0.346 mmol, 142 mg, 1.0 equiv) in CH_2_Cl_2_ (8 mL) was added to the again cooled solution. The mixture was then stirred for 19 h at ambient temperature. It was subsequently diluted with water (50 mL) and extracted with CH_2_Cl_2_ (3 × 50 mL). The combined organic layers were dried (MgSO_4_), filtered and all volatile materials were removed under reduced pressure. The residue was chromatographed (SiO_2_, hexanes/EtOAc 6:1, *R*_f_ = 0.25) to yield compound **3** (148 mg, 0.247 mmol, 71%) as a bright yellow solid. Mp 86–88 °C; ^1^H NMR (500 MHz, CDCl_3_) δ 1.31 (t, *J* = 7.1 Hz, 3H), 1.41 (t, *J* = 7.1 Hz, 3H), 1.46–1.59 (m, 2H), 1.66–1.81 (m, 4H), 1.91 (dq, *J* = 13.6 Hz, *J* = 7.0 Hz, 1H), 2.37–2.48 (m, 3H), 3.07–3.12 (m, 1H), 3.14–3.19 (m, 1H), 3.54–3.60 (m, 1H), 4.29 (q, *J* = 7.1 Hz, 2H), 4.37 (q, *J* = 7.1 Hz, 2H), 4.51 (d, *J* = 5.3 Hz, 2H), 7.18 (s, 1H), 7.48 (d, *J* = 8.1 Hz, 2H), 7.59 (d, *J* = 8.1 Hz, 2H), 8.03 (t, *J* = 5.3 Hz, 1H), 9.21–9.22 (m, 1H), 10.40–10.42 (m, 1H) ppm; ^13^C{^1^H} NMR (125 MHz, CDCl_3_) δ 14.11 (CH_3_), 14.42 (CH_3_), 25.40 (CH_2_), 28.98 (CH_2_), 34.81 (CH_2_), 38.25 (CH_2_), 38.60 (CH_2_), 40.35 (CH_2_), 47.11 (CH_2_), 56.52 (CH), 61.30 (CH_2_), 61.81 (CH_2_), 113.49 (CH), 115.62 (C), 121.26 (C), 124.20 (CH), 124.26 (q, *J* = 271.9 Hz, C), 125.79 (q, *J* = 3.4 Hz, 2 CH), 127.50 (2 CH), 129.73 (C), 129.76 (q, *J* = 32.3 Hz, C), 143.18 (C), 145.52 (C), 167.56 (C), 168.09 (C), 171.21 (C) ppm; IR (ATR): 3387 (w), 3304 (w), 2981 (w), 2932 (w), 1715 (m), 1680 (s), 1657 (m), 1621 (w), 1543 (s), 1500 (w), 1473 (w), 1448 (w), 1415 (m), 1367 (w), 1329 (s), 1264 (m), 1216 (vs), 1160 (s), 1108 (s), 1094 (s), 1068 (s), 1019 (m), 912 (w), 896 (w), 866 (w), 827 (m), 791 (m), 667 (w), 633 (w), 588 (w), 577 (w) cm^−1^; MS (EI, 70 eV) *m/z* (%): 598 (18) [M^+^], 551 (26), 418 (16), 405 (30), 158 (55), 73 (100), 66 (24); HRMS (EI): [M^+^] calcd for C_28_H_33_F_3_N_2_O_5_S_2_^+^, 598.1777; found, 598.1776; UV–vis (CH_2_Cl_2_): λ_max_ (lg ε) = 420 nm (3.67); fluorescence (CH_2_Cl_2_): λ_em_ = 495 nm, λ_ex_ = 420 nm, Φ = 0.57; C_28_H_33_F_3_N_2_O_5_S_2_ (598.71 g·mol^−1^).

**Diethyl 2-[3-(allyloxycarbonylamino)propylamino]-5-[4-(trifluoromethyl)benzylamino]terephthalate (6):** A solution of DAT **5** (0.254 mmol, 100 mg, 1.0 equiv) in CH_2_Cl_2_ (2 mL) was added to a cooled (ice-water bath) suspension of 4-(trifluoromethyl)benzaldehyde (0.38 mmol, 66 mg, 1.5 equiv) in CH_2_Cl_2_ (2 mL). After stirring the mixture for 15 min at ambient temperature, it was cooled again (ice-water bath) and ZnCl_2_ (0.13 mmol, 18 mg, 0.5 equiv) and after 30 min NaBH_3_CN (0.38 mmol, 66 mg, 1.5 equiv) were added and the resulting mixture was further stirred at ambient temperature for 19 h. It was then diluted with water (25 mL) and extracted with CH_2_Cl_2_ (2 × 25 mL). The combined organic layers were dried (MgSO_4_) and evaporated after filtration. Chromatography of the residue (SiO_2_, hexanes/EtOAc 3:1, *R*_f_ = 0.24) furnished title compound **6** (128 mg, 0.232 mmol, 91%) as an orange-red solid. Mp 127–128 °C; ^1^H NMR (500 MHz, CDCl_3_) δ 1.28 (t, *J* = 7.1 Hz, 3H), 1.41 (t, *J* = 7.1 Hz, 3H), 1.88 (pent, *J* = 6.7 Hz, 2H), 3.23 (t, *J* = 6.6 Hz, 2H), 3.33 (q, *J* = 6.3 Hz, 2H), 4.24 (q, *J* = 7.1 Hz, 2H), 4.37 (q, *J* = 7.1 Hz, 2H), 4.46 (s, 2H), 4.35 (d, *J* = 4.6 Hz, 2H), 4.89 (br.s, 1H), 5.19 (d, *J* = 10.4 Hz, 1H), 5.29 (dd, *J* = 17.2 Hz, *J* = 1.4 Hz, 1H), 5.91 (ddt, *J* = 16.9 Hz, *J* = 10.7 Hz, *J* = 5.6 Hz, 1H), 6.80 (br. s, 1H), 7.18 (s, 1H), 7.30 (s, 1H), 7.38 (br. s, 1H), 7.49 (d, *J* = 8.0 Hz, 2H), 7.58 (d, *J* = 8.1 Hz, 2H) ppm; ^13^C{^1^H} NMR (125 MHz, CDCl_3_) δ 14.22 (CH_3_), 14.46 (CH_3_), 29.72 (CH_2_), 39.19 (CH_2_), 41.13 (CH_2_), 47.72 (CH_2_), 60.83 (CH_2_), 61.07 (CH_2_), 65.63 (CH_2_), 114.15 (CH), 114.81 (CH), 116.88 (C), 117.52 (C), 117.68 (CH), 124.35 (q, *J* = 271.9 Hz, C), 125.64 (q, *J* = 3.5 Hz, 2 CH), 127.60 (2 CH), 123.49 (q, *J* = 32.3 Hz, C), 133.09 (C), 140.59 (C), 141.65 (C), 144.07 (C), 156.44 (C), 167.87 (C), 168.08 (C) ppm; IR (ATR): 3378 (w), 3323 (w), 2992 (w), 2942 (w), 2882 (w), 1676 (s), 1623 (w), 1529 (s), 1475 (w), 1457 (w), 1420 (m), 1392 (w), 1368 (w), 1331 (m), 1267 (w), 1203 (vs), 1159 (m), 1106 (vs), 1070 (s), 1022 (m), 940 (w), 921 (w), 875 (w), 860 (w), 826 (w), 790 (m), 631 (w), 602 (w) cm^−1^; MS (EI, 70 eV) *m/z* (%): 551 (86) [M^+^], 493 (100), 423 (12), 376 (62), 334 (35), 244 (13), 158 (30); HRMS (EI): [M^+^] calcd for C_27_H_32_F_3_N_3_O_6_^+^, 551.2238; found, 551.2236; UV–vis (CH_2_Cl_2_): λ_max_ (lg ε) = 473 nm (3.83); fluorescence (CH_2_Cl_2_): λ_em_ = 567 nm, λ_ex_ = 473 nm, Φ = 0.12; C_27_H_32_F_3_N_3_O_6_ (551.56 g·mol^−1^).

**Diethyl 2-[(3-aminopropyl)amino]-5-[4-(trifluoromethyl)benzylamino]terephthalate (8):** Under exclusion of air and moisture, morpholine (1.17 mmol, 102 mg, 5.0 equiv) was added to a solution of carbamate **6** (0.234 mmol, 129 mg, 1.0 equiv) in abs. CH_2_Cl_2_ (3 mL). The mixture was degassed (three cycles of freeze, pump and thaw). Then Pd(PPh_3_)_4_ (12 µmol, 14 mg, 0.05 equiv) was added and the mixture was stirred for 1 h at ambient temperature under an inert atmosphere. After adding charcoal (2 mg), it was stirred for 5 min at ambient temperature and then filtered. The filtrate was evaporated and the residue chromatographed (SiO_2_, EtOAc/MeOH 6:1 with 1 vol % NEt_3_, *R*_f_ = 0.15) to furnish title compound **8** (104 mg, 0.222 mmol, 95%) as red solid. Mp 189–191 °C; ^1^H NMR (500 MHz, DMSO-*d*_6_) δ 1.18 (t, *J* = 7.1 Hz, 3H), 1.33 (t, *J* = 7.1 Hz, 3H), 1.71 (pent, *J* = 6.8 Hz, 2H), 2.70 (t, *J* = 6.9 Hz, 2H), 3.51 (q, *J* = 6.2 Hz, 2H), 4.16 (q, *J* = 7.1 Hz, 2H), 4.33 (q, *J* = 7.1 Hz, 2H), 4.48 (d, *J* = 5.7 Hz, 2H), 6.68 (t, *J* = 4.6 Hz, 1H), 7.08 (s, 1H), 7.24 (s, 1H), 7.33 (t, *J* = 5.8 Hz, 1H), 7.57 (d, *J* = 8.1 Hz, 2H), 7.69 (d, *J* = 8.2 Hz, 2H) ppm, signals for two NH protons were not detected; ^13^C{^1^H} NMR (125 MHz, DMSO-*d*_6_) δ 13.81 (CH_3_), 14.12 (CH_3_), 30.54 (CH_2_), 38.45 (CH_2_), 40.24 (CH_2_), 46.48 (CH_2_), 60.49 (CH_2_), 60.79 (CH_2_), 113.42 (CH), 114.35 (CH), 115.84 (C), 117.06 (C), 124.39 (q, *J* = 271.8 Hz, C), 125.30 (q, *J* = 3.5 Hz, 2 CH), 127.37 (2 CH), 127.29 (q, *J* = 31.6 Hz, C), 139.46 (C), 140.80 (C), 145.07 (C), 166.88 (C), 167.04 (C) ppm; IR (ATR): 3392 (m), 3250 (br), 2907 (br), 2751 (w), 2680 (w), 2600 (w), 2522 (w), 1669 (s), 1621 (w), 1530 (s), 1474 (w), 1418 (w), 1390 (w), 1368 (w), 1338 (m), 1310 (w), 1276 (m), 1215 (vs), 1152 (s), 1106 (vs), 1070 (s), 1043 (w), 1019 (m), 961 (m), 879 (w), 828 (w), 786 (m), 748 (w), 605 (w), 576 (w) cm^−1^; MS (EI, 70 eV) *m/z* (%): 467 (100) [M^+^], 423 (21), 410 (16), 376 (50), 232 (18), 158 (49); HRMS (EI): [M^+^] calcd for C_23_H_28_F_3_N_3_O_4_^+^, 467.2026; found, 467.2012; UV–vis (MeOH): λ_max_ (lg ε) = 464 nm (3.58); fluorescence (MeOH): λ_em_ = 566 nm, λ_ex_ = 464 nm, Φ = 0.09; C_23_H_28_F_3_N_3_O_4_ (467.49 g·mol^−1^).

***rac*-Diethyl 2-{3-[5-(1,2-dithiolan-3-yl)pentanoylamino]propylamino}-5-[4-(trifluoromethyl)benzylamino]terephthalate (7): ***rac*-α-Lipoic acid (0.21 mmol, 44 mg, 2.0 equiv) and COMU (0.320 mmol, 137 mg, 3.0 equiv) were successively added to a solution of DIPEA (0.32 mmol, 41 mg, 3.0 equiv) in CH_2_Cl_2_ (2 mL) and the solution was stirred for 1 h at ambient temperature. The solution was added dropwise to a suspension of amine **8** (107 µmol, 50.0 mg, 1.0 equiv) in CH_2_Cl_2_ (2 mL) and the mixture was stirred for 16 h at ambient temperature. It was diluted with water (25 mL) and the mixture was extracted with CH_2_Cl_2_ (2 × 25 mL). The combined organic layers were dried (MgSO_4_), filtered and all volatile materials were removed under reduced pressure. The residue was chromatographed (SiO_2_, hexanes/EtOAc 1:2, *R*_f_ = 0.23) to yield compound **7** (48 mg, 73 µmol, 68%) as a red resin. ^1^H NMR (500 MHz, CDCl_3_) δ 1.28 (t, *J* = 7.1 Hz, 3H), 1.33–1.48 (m, 2H), 1.41 (t, *J* = 7.1 Hz, 3H), 1.59–1.73 (m, 4H), 1.85–1.93 (m, 3H), 2.17 (dt, *J* = 7.6 Hz, *J* = 1.6 Hz, 2H), 2.40–2.49 (m, 1H), 3.07–3.14 (m, 1H), 3.15–3.18 (m, 1H), 3.23 (t, *J* = 6.5 Hz, 2H), 3.40 (q, *J* = 6.5 Hz, 2H), 3.52–3.60 (m, 1H), 4.24 (q, *J* = 7.1 Hz, 2H), 4.37 (q, *J* = 7.1 Hz, 2H), 4.46 (s, 2H), 5.74 (t, *J* = 5.3 Hz, 1H), 7.18 (s, 1H), 7.30 (s, 1H), 7.50 (d, *J* = 8.1 Hz, 2H), 7.58 (d, *J* = 8.1 Hz, 2H) ppm, signals for three NH-protons could not be detected; ^13^C{^1^H} NMR (125 MHz, CDCl_3_) δ 14.26 (CH_3_), 14.51 (CH_3_), 25.53 (CH_2_), 29.06 (CH_2_), 29.30 (CH_2_), 34.77 (CH_2_), 36.65 (CH_2_), 38.03 (CH_2_), 38.61 (CH_2_), 40.37 (CH_2_), 41.73 (CH_2_), 47.71 (CH_2_), 56.54 (CH), 60.92 (CH_2_), 61.14 (CH_2_), 114.22 (CH), 114.80 (CH), 116.91 (C), 117.50 (C), 124.34 (q, *J* = 272.3 Hz, C), 125.66 (q, *J* = 3.6 Hz, 2 CH), 127.60 (2 CH), 129.49 (q, *J* = 32.2 Hz, C), 140.68 (C), 141.56 (C), 144.02 (C), 167.93 (C), 168.07 (C), 172.99 (C) ppm; IR (ATR): 2980 (w), 2933 (w), 2866 (w), 1726 (w), 1682 (m), 1618 (w), 1607 (w), 1575 (w), 1529 (m), 1463 (w), 1418 (w), 1371 (w), 1325 (m), 1258 (w), 1216 (m), 1195 (s), 1164 (m), 1118 (s), 1104 (s), 1083 (s), 1065 (m), 1017 (w), 993 (w), 839 (vs), 790 (m), 739 (w), 632 (w), 608 (w) cm^−1^; HRMS (ESI, pos. mode): [M + H^+^] calcd for C_31_H_41_F_3_N_3_O_5_S_2_^+^, 656.2434; found, 656.2440; UV–vis (CH_2_Cl_2_): λ_max_ (lg ε) = 514 nm (3.58); fluorescence (CH_2_Cl_2_): λ_em_ = 514 nm, λ_ex_ = 566 nm, Φ = 0.04; C_31_H_40_F_3_N_3_O_5_S_2_ (655.79 g·mol^−1^).

**Preparation of SAMs of compounds 3 and 7:** Gold surfaces were prepared onto cleaned glass slides as the support by depositing 0.5 nm of chromium by electron-beam evaporation as adhesion layer and 200 nm of gold by resistive heating using an evaporation chamber (minicoater, Tectra GmbH, Frankfurt, Germany). The thickness was monitored by means of a quartz crystal microbalance (Tectra GmbH, Frankfurt, Germany). The gold substrates were freshly prepared prior to each experiment. Compound **3** or compound **7** were dissolved in 20 mL ethanol (analytical grade, Fisher Chemicals) and diluted to a final concentration of 3 × 10^–4^ mol·L^–1^. The gold substrates were immersed in the ethanolic solutions of compound **3** or compound **7** immediately after preparation of the gold layer. The gold substrates were left in the ethanolic solution for self-assembly over 24 h, then removed and rinsed with copious amounts of ethanol (analytical grade, Fisher Chemicals) and eventually dried in an argon stream.

## Supporting Information

File 1 Copies of NMR spectra of all reported compounds.

## References

[1] Liebermann H (1914). Justus Liebigs Ann Chem.

[2] Christoffers J (2018). Eur J Org Chem.

[3] Shimizu M, Asai Y, Takeda Y, Yamatani A, Hiyama T (2011). Tetrahedron Lett.

[4] Shimizu M, Fukui H, Natakani M, Sakaguchi H (2016). Eur J Org Chem.

[5] Tang B, Wang C, Wang Y, Zhang H (2017). Angew Chem, Int Ed.

[6] Sinnreich J (1980). Synthesis.

[7] Zhang Y, Starynowicz P, Christoffers J (2008). Eur J Org Chem.

[8] Pflantz R, Christoffers J (2009). Chem – Eur J.

[9] Freimuth L, Christoffers J (2015). Chem – Eur J.

[10] Christoffers J, Freimuth L, Rozzi C, Lienau C (2015). Synthesis.

[11] Wache N, Schröder C, Koch K-W, Christoffers J (2012). ChemBioChem.

[12] Wache N, Scholten A, Klüner T, Koch K-W, Christoffers J (2012). Eur J Org Chem.

[13] Wallisch M, Sulmann S, Koch K-W, Christoffers J (2017). Chem – Eur J.

[14] Jewett J C, Bertozzi C R (2010). Chem Soc Rev.

[15] Wu D, Cheung S, Devocelle M, Zhang L-J, Chen Z-L, O'Shea D F (2015). Chem Commun.

[16] Haimi P, Sikorskaite-Gudziuniene S, Baniulis D (2015). Proteomics.

[17] Dietz L, Bosque A, Pankert P, Ohnesorge S, Merz P, Anel A, Schnölzer M, Thierse H-J (2009). Proteomics.

[18] Ulman A (1996). Chem Rev.

[19] Gooding J J, Mearns F, Yang W, Liu J (2003). Electroanalysis.

[20] Wilbur J L, Kumar A, Biebuyck H A, Kim E, Whitesides G M (1996). Nanotechnology.

[21] Ahn Y, Hong S, Jang J (2006). J Phys Chem B.

[22] Li Y, Yuan B, Ji H, Han D, Chen S, Tian F, Jiang X (2007). Angew Chem, Int Ed.

[23] Jadhav S A (2012). J Mater Chem.

[24] Zhang X, Shi F, Niu J, Jiang Y, Wang Z (2008). J Mater Chem.

[25] Laibinis P E, Whitesides G M, Allara D L, Tao Y-T, Parikh A N, Nuzzo R G (1991). J Am Chem Soc.

[26] Smalley J F, Finklea H O, Chidsey C E D, Linford M R, Creager S E, Ferraris J P, Chalfant K, Zawodzinsk T, Feldberg S W, Newton M D (2003). J Am Chem Soc.

[27] Eckermann A L, Feld D J, Shaw J A, Meade T J (2010). Coord Chem Rev.

[28] Bardea A, Katz E, Willner I (2000). Electroanalysis.

[29] Beulen M W J, van Veggel F C J M, Reinhoudt D N (1999). Chem Commun.

[30] Hickman J J, Ofer D, Laibinis P E, Whitesides G M, Wrighton M S (1991). Science.

[31] Yang W, Gooding J J, Hibbert D B (2001). Analyst.

[32] Yang W, Jaramillo D, Gooding J J, Hibbert D B, Zhang R, Willett G D, Fisher K J (2001). Chem Commun.

[33] Yang W, Gooding J J, Hibbert D B (2001). J Electroanal Chem.

[34] Gooding J J, Erokhin P, Losic D, Yang W, Policarpio V, Liu J, Ho F M, Situmorang M, Hibbert D B, Shapter J G (2001). Anal Sci.

[35] Gooding J J, Hibbert D B (1999). TrAC, Trends Anal Chem.

[36] Yousaf M N, Mrksich M (1999). J Am Chem Soc.

[37] Zhao C, Witte I, Wittstock G (2006). Angew Chem, Int Ed.

[38] Lesch A, Vaske B, Meiners F, Momotenko D, Cortés-Salazar F, Girault H H, Wittstock G (2012). Angew Chem, Int Ed.

[39] Gooding J J, Ciampi S (2011). Chem Soc Rev.

[40] Scofield J H (1976). J Electron Spectrosc Relat Phenom.

[41] Wu Z-Q, Jiang X-K, Zhu S-Z, Li Z-T (2004). Org Lett.

[42] Penning M, Christoffers J (2012). Eur J Org Chem.

[43] Hjelmgaard T, Faure S, Staerk D, Taillefumier C, Nielsen J (2011). Eur J Org Chem.

[44] Buschbeck L, Christoffers J (2018). J Org Chem.

[45] Jimmidi R, Shroff G K, Satyanarayana M, Reddy B R, Kapireddy J, Sawant M A, Sitaswad S L, Arya P, Mitra P (2014). Eur J Org Chem.

[46] Pachamuthu K, Zhu X, Schmidt R R (2005). J Org Chem.

[47] Parker C A, Rees W T (1960). Analyst.

[48] Demas J N, Crosby G A (1971). J Phys Chem.

[49] Fery-Forgues S, Lavabre D (1999). J Chem Educ.

[50] Snare M J, Treloar F E, Ghiggino K P, Thistlethwaite P J (1982). J Photochem.

[51] Casey K G, Quitevis E L (1988). J Phys Chem.

[52] Capitao D, Sahli R, Raouafi N, Limoges B, Fave C, Schöllhorn B (2016). ChemElectroChem.

[53] Desimoni E, Brunetti B (2015). Chemosensors.

[54] Gardella J A, Ferguson S A, Chin R L (1986). Appl Spectrosc.

[55] Chinwangso P, Lee H J, Lee T R (2015). Langmuir.

[56] López G P, Castner D G, Ratner B D (1991). Surf Interface Anal.

[57] Spitzer A, Lüth H (1985). Surf Sci.

[58] Zhang X, Ptasinska S (2014). J Phys Chem C.

[59] Duwez A-S (2004). J Electron Spectrosc Relat Phenom.

[60] Laibinis P E, Graham R L, Biebuyck H A, Whitesides G M (1991). Science.

[61] Graham R L, Bain C D, Biebuyck H A, Laibinis P E, Whitesides G M (1993). J Phys Chem.

[62] Heister K, Zharnikov M, Grunze M, Johansson L S O, Ulman A (2001). Langmuir.

[63] Han M G, Im S S (2000). Polymer.

