# Supplementary material for: Diaminoterephthalate–α-lipoic acid conjugates with fluorinated residues
Source: Beilstein J Org Chem. 2019 Apr 26;15:981–91. doi: 10.3762/bjoc.15.96 (PMC6541350; doi:10.3762/bjoc.15.96)
Supplement: File 1 — Copies of NMR spectra of all reported compounds. [file Beilstein_J_Org_Chem-15-981-s001.pdf]

BEILSTEIN JOURNAL OF ORGANIC CHEMISTRY

Supporting Information

for

Diaminoterephthalate–α-lipoic acid conjugates with fluorinated residues

Leon Buschbeck, Aleksandra Markovic, Gunther Wittstock and Jens Christoffers

Beilstein J. Org. Chem. **2019,** *15,* 981–991. doi:10.3762/bjoc.15.96

Copies of NMR spectra of all reported compounds

License and Terms: This is a supporting information file under the terms of the Creative Commons Attribution License (http://creativecommons.org/licenses/by/4.0). Please note that the reuse, redistribution and reproduction in particular requires that the authors and source are credited.
The license is subject to the *Beilstein Journal of Organic Chemistry* terms and conditions: (https://www.beilstein-journals.org/bjoc)

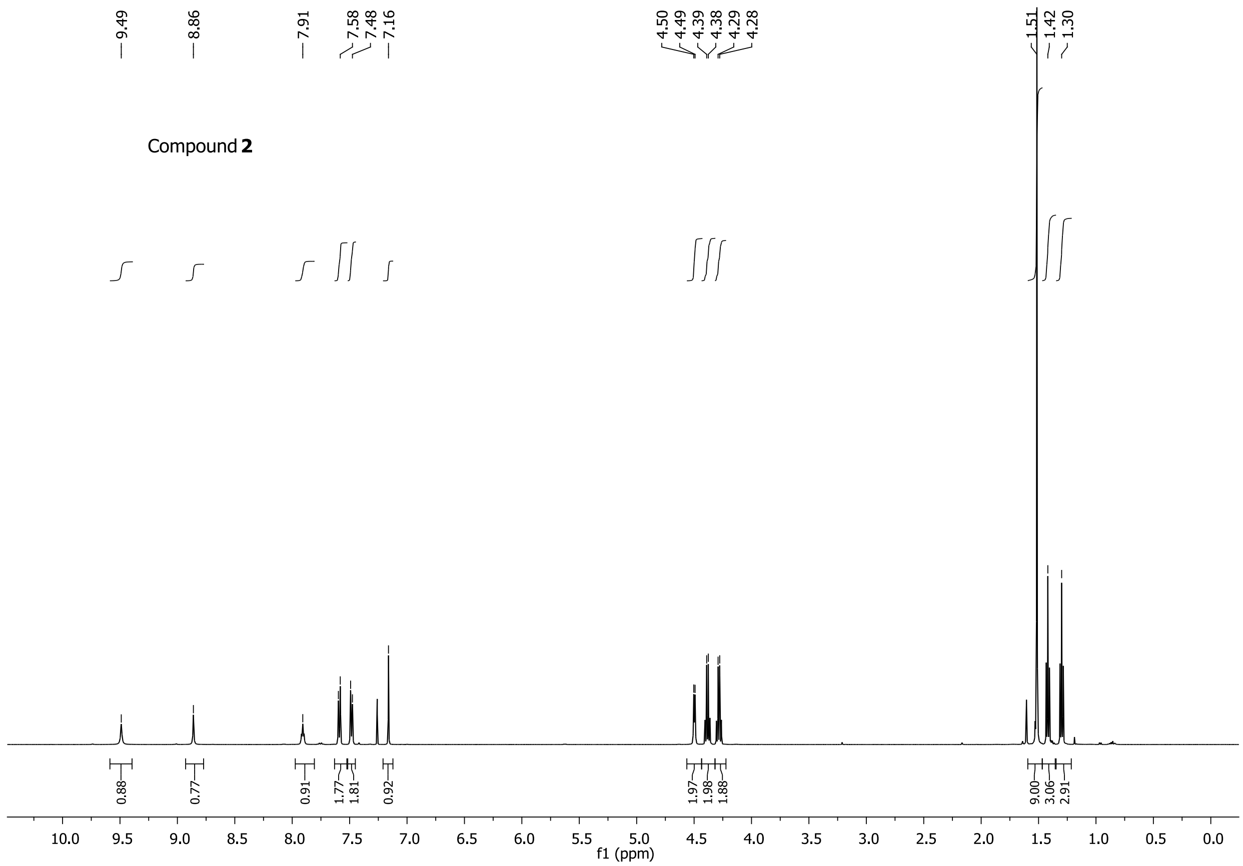

9.49
8.86
7.91
7.58
7.48
7.16
4.50
4.49
4.39
4.38
4.29
4.28
1.51
1.42
1.30
Compound **2**
0.88
0.77
0.91
1.77
1.81
0.92
1.97
1.98
1.88
9.00
3.06
2.91
10.0 9.5 9.0 8.5 8.0 7.5 7.0 6.5 6.0 5.5 5.0 4.5 4.0 3.5 3.0 2.5 2.0 1.5 1.0 0.5 0.0
f1 (ppm)

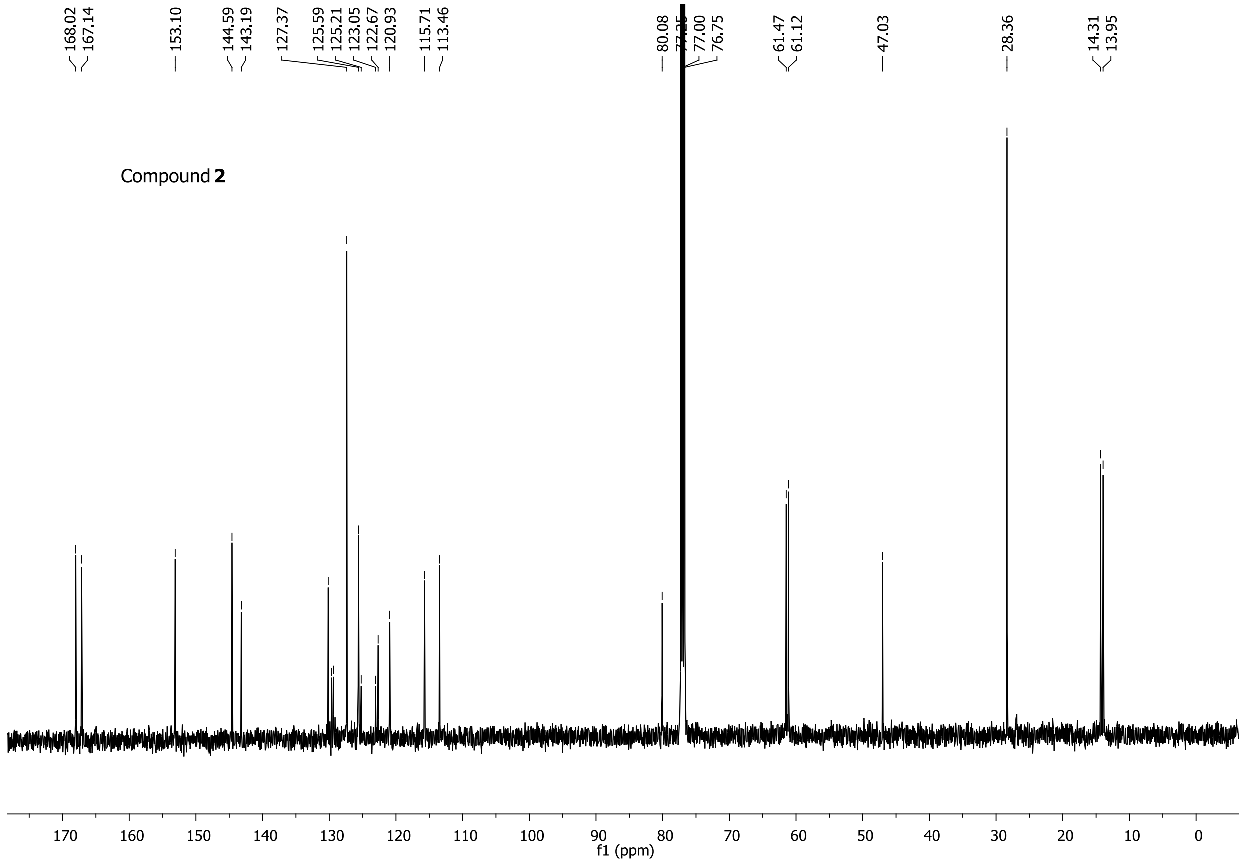

168.02
167.14
153.10
144.59
143.19
127.37
125.59
125.21
123.05
122.67
120.93
115.71
113.46
80.08
77.00
76.75
61.47
61.12
47.03
28.36
14.31
13.95
Compound **2**
170 160 150 140 130 120 110 100 90 80 70 60 50 40 30 20 10 0
f1 (ppm)

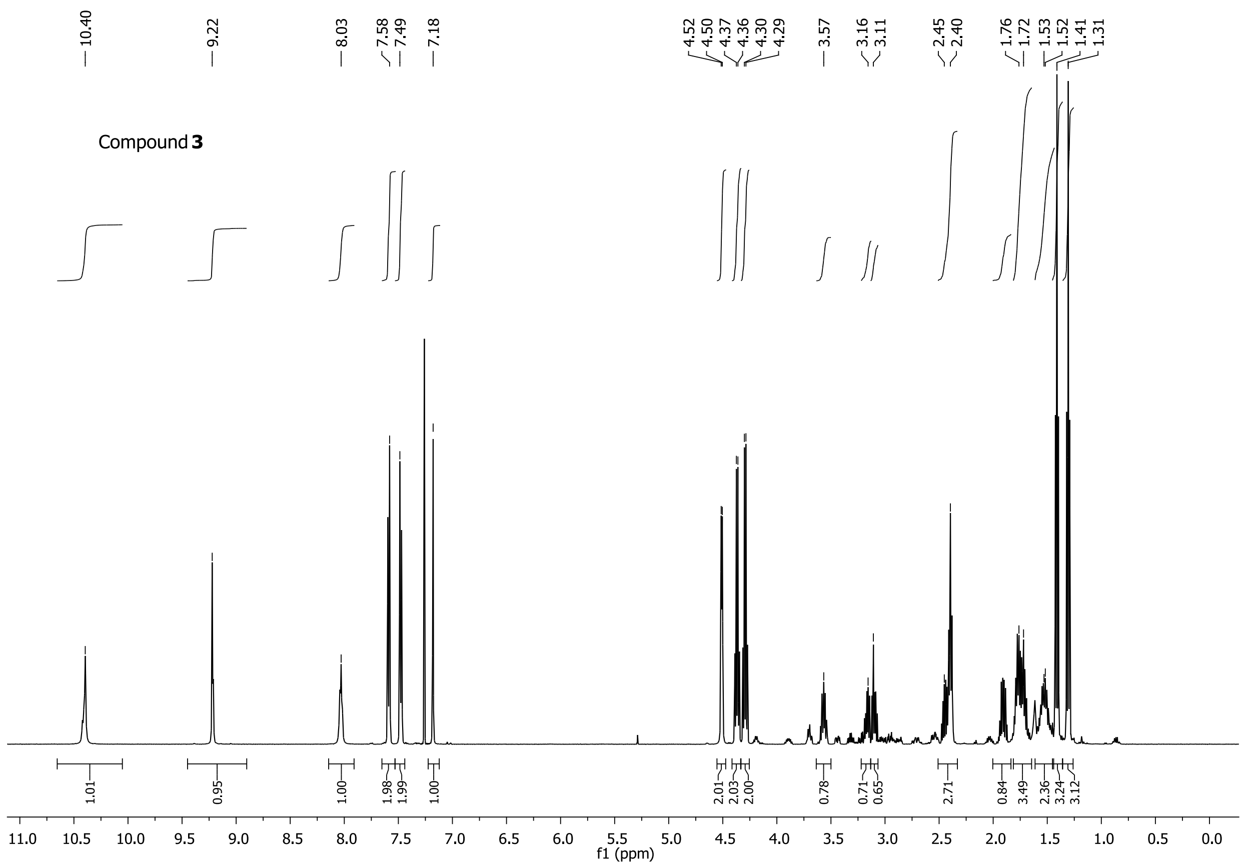
10.40
9.22
8.03
7.58
7.49
7.18
4.52
4.50
4.37
4.36
4.30
4.29
3.57
3.16
3.11
2.45
2.40
1.76
1.72
1.53
1.52
1.41
1.31
Compound **3**
f1 (ppm)

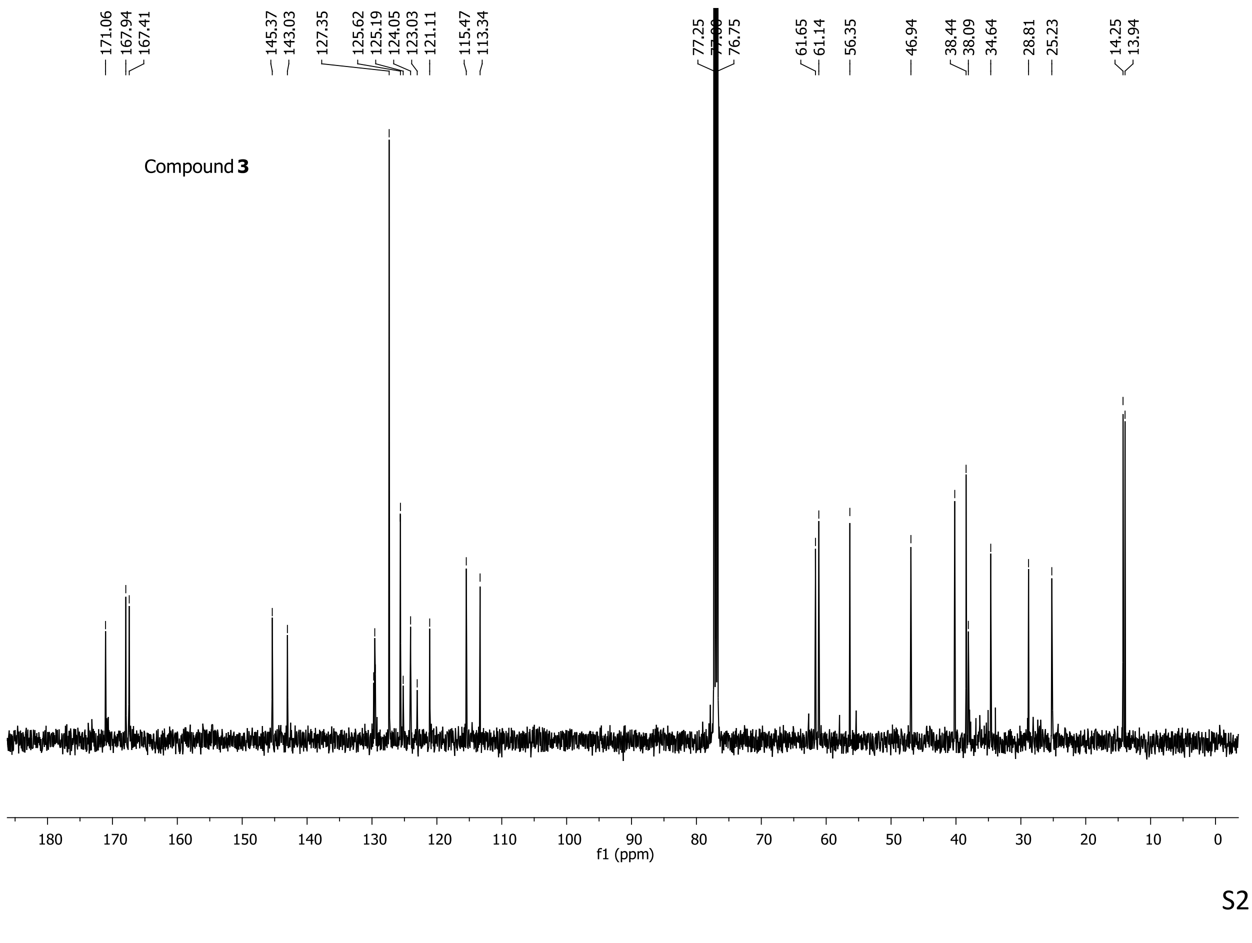
171.06
167.94
167.41
145.37
143.03
127.35
125.62
125.19
124.05
123.03
121.11
115.47
113.34
77.25
76.75
61.65
61.14
56.35
46.94
38.44
38.09
34.64
28.81
25.23
14.25
13.94
Compound **3**
f1 (ppm)

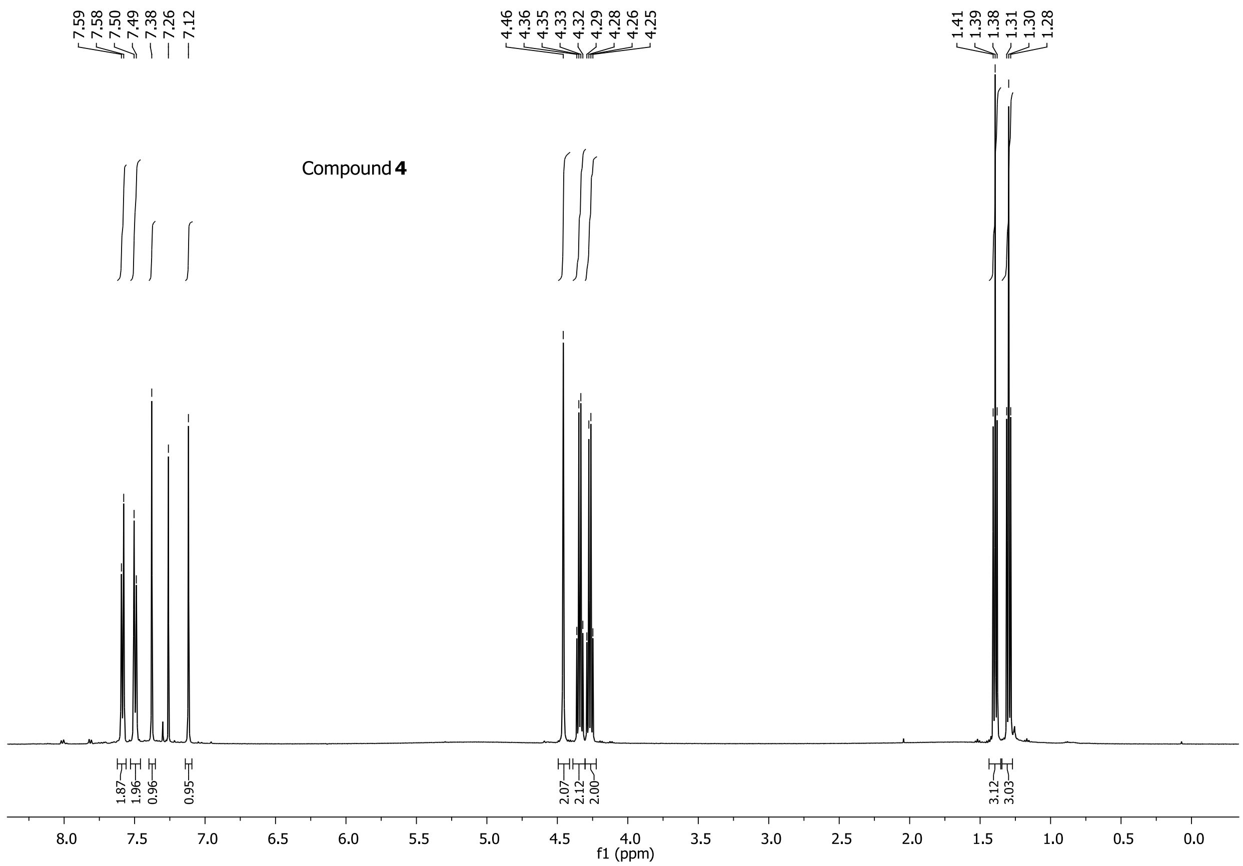
7.59
7.58
7.50
7.49
7.38
7.26
7.12
4.46
4.36
4.35
4.33
4.32
4.29
4.28
4.26
4.25
1.41
1.39
1.38
1.31
1.30
1.28
Compound 4
1.87
1.96
0.96
0.95
2.07
2.12
2.00
3.12
3.03
8.0
7.5
7.0
6.5
6.0
5.5
5.0
4.5
4.0
3.5
3.0
2.5
2.0
1.5
1.0
0.5
0.0
f1 (ppm)

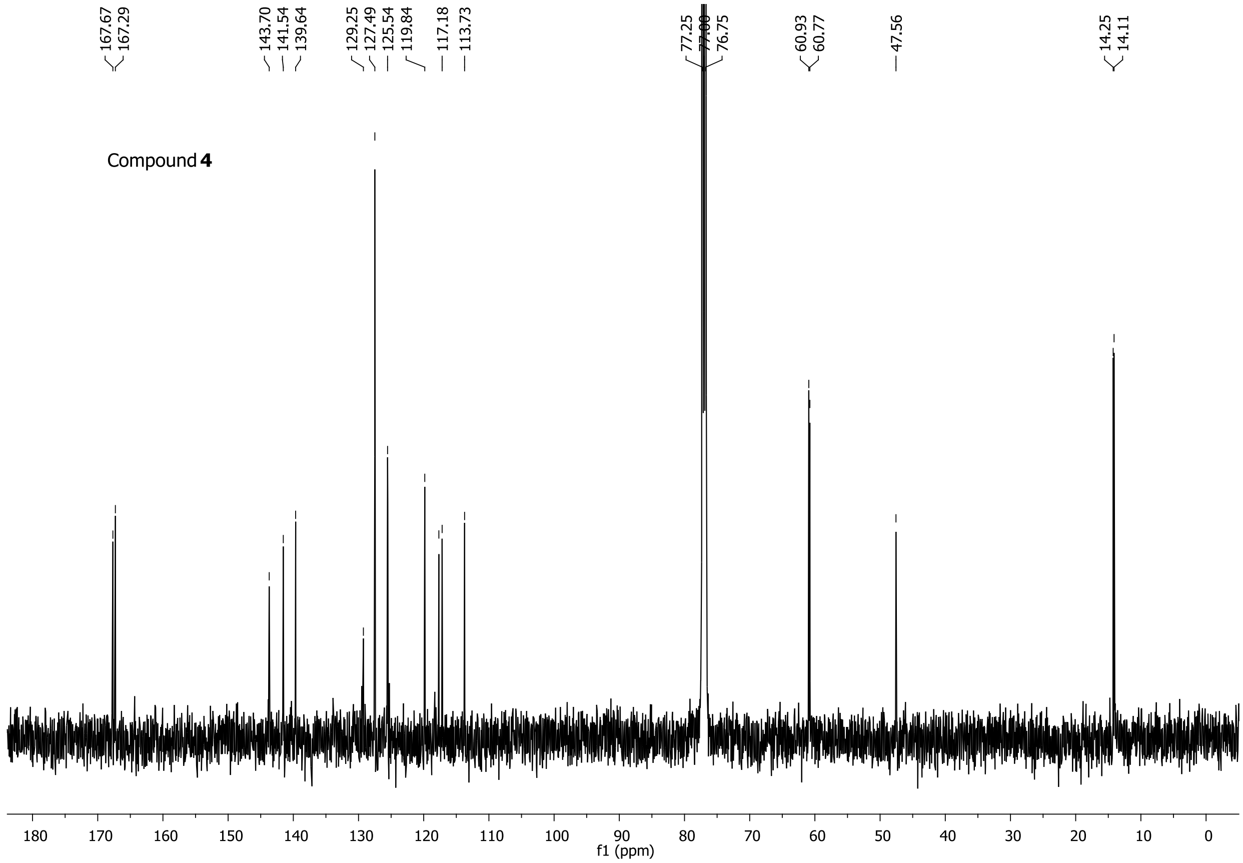
167.67
167.29
143.70
141.54
139.64
129.25
127.49
125.54
119.84
117.18
113.73
77.25
76.75
60.93
60.77
47.56
14.25
14.11
Compound 4
180
170
160
150
140
130
120
110
100
90
80
70
60
50
40
30
20
10
0
f1 (ppm)

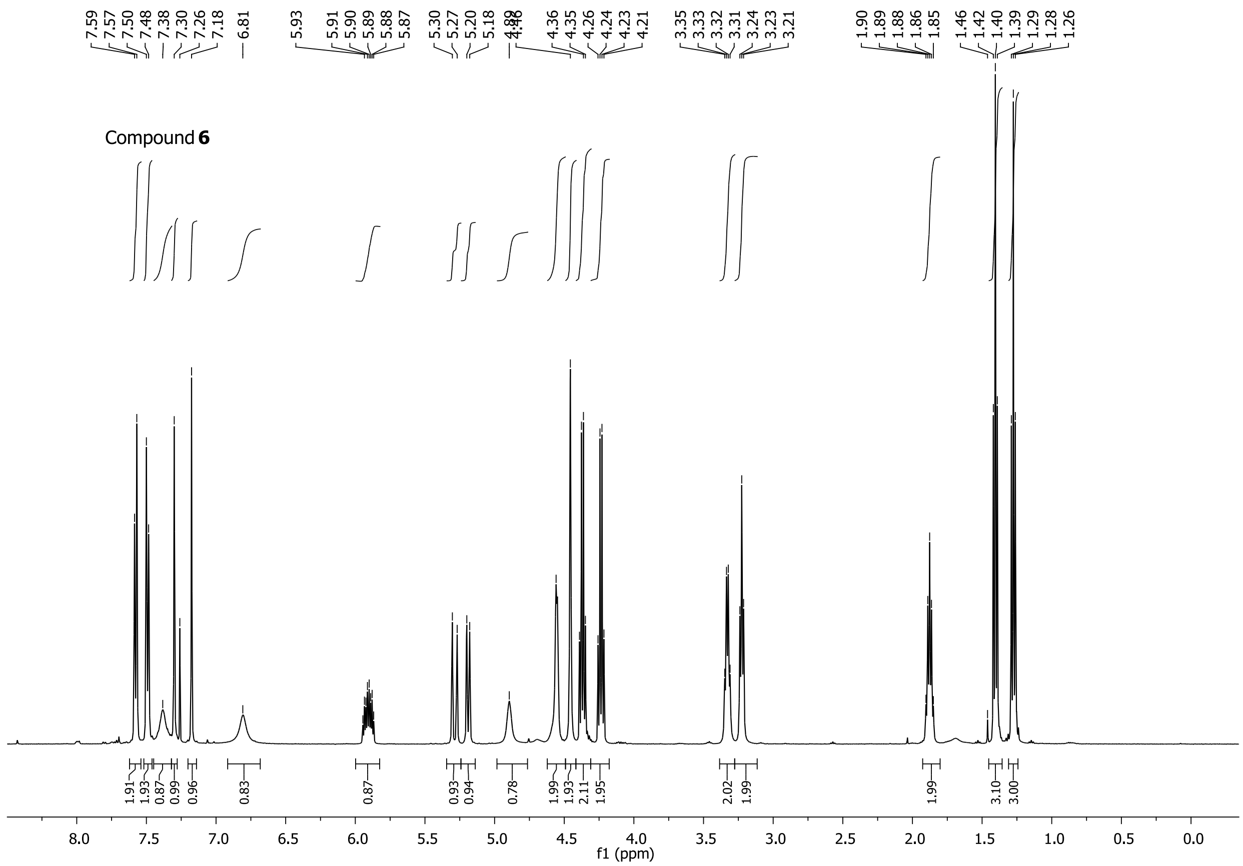
Compound 6
f1 (ppm)

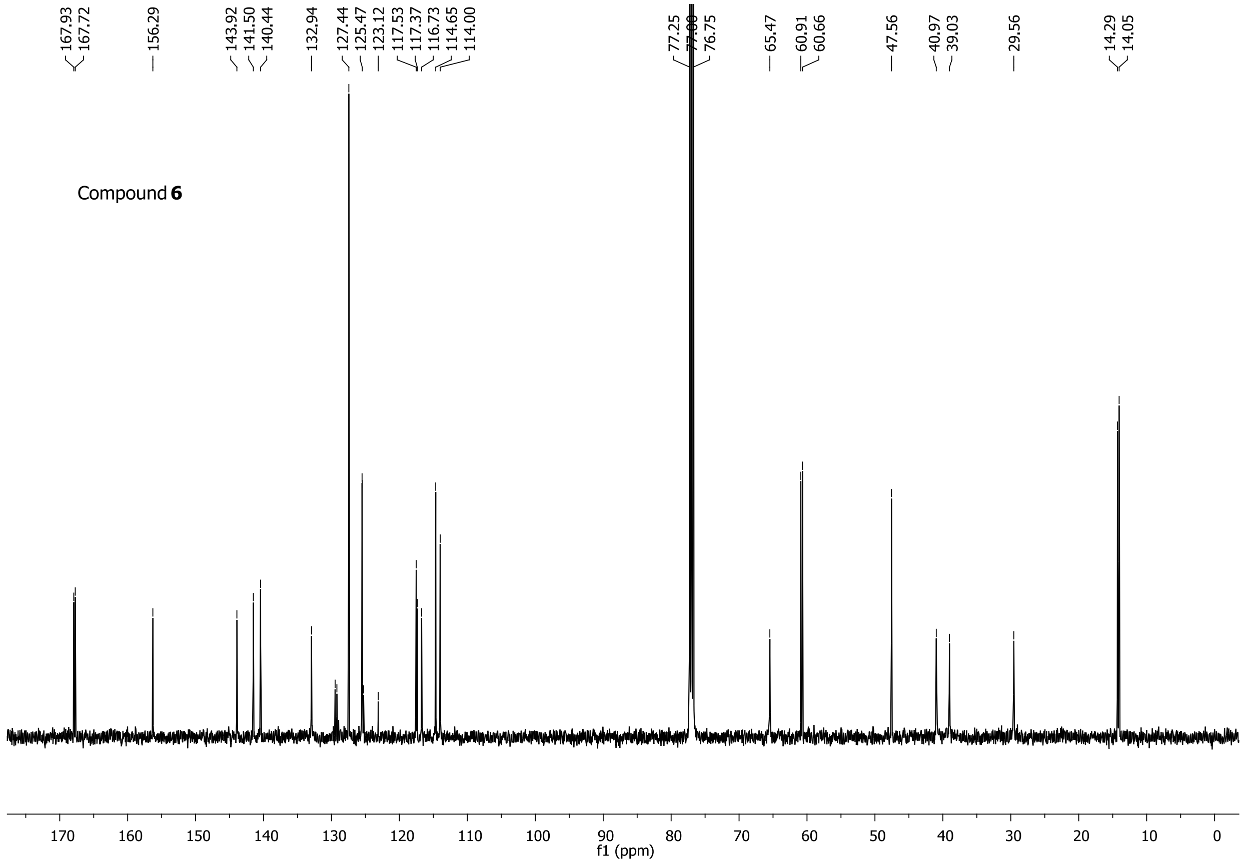
Compound 6
f1 (ppm)

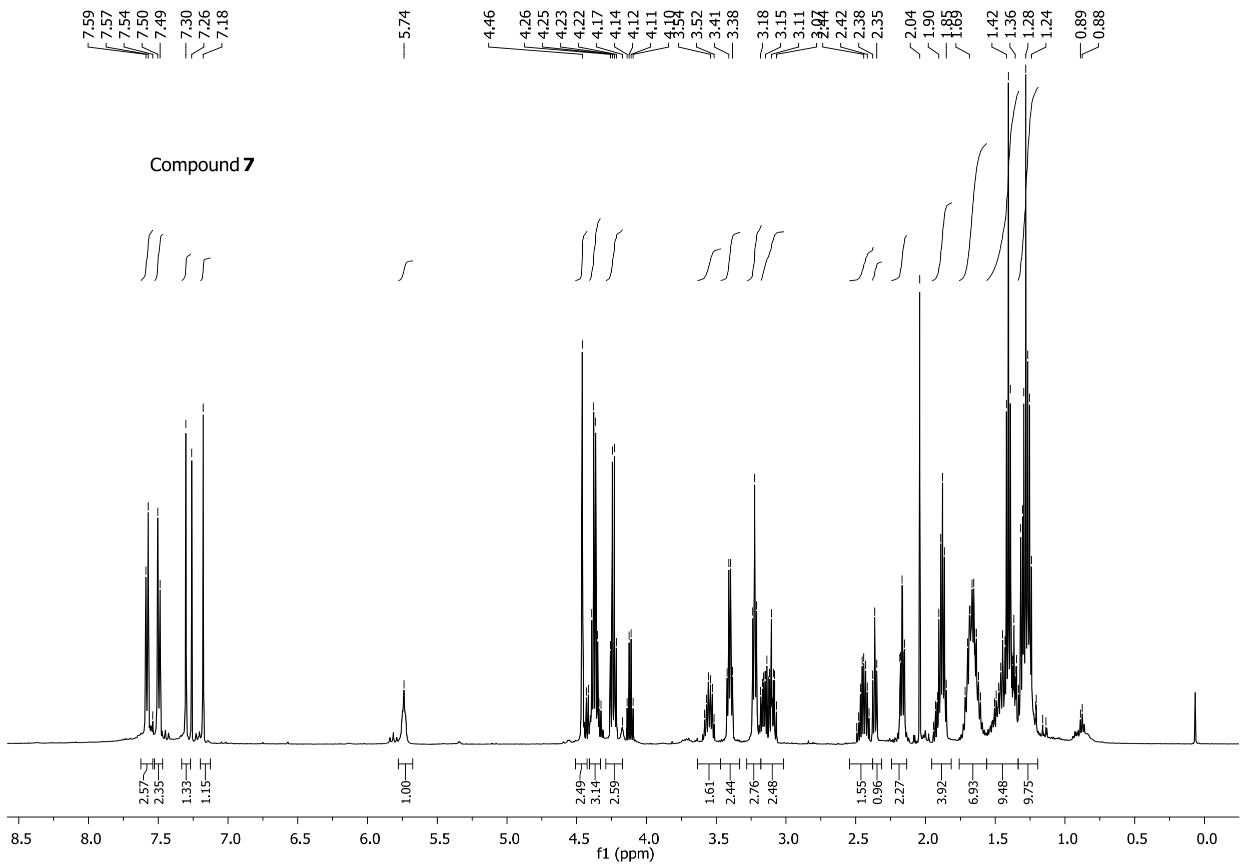
Compound 7
f1 (ppm)

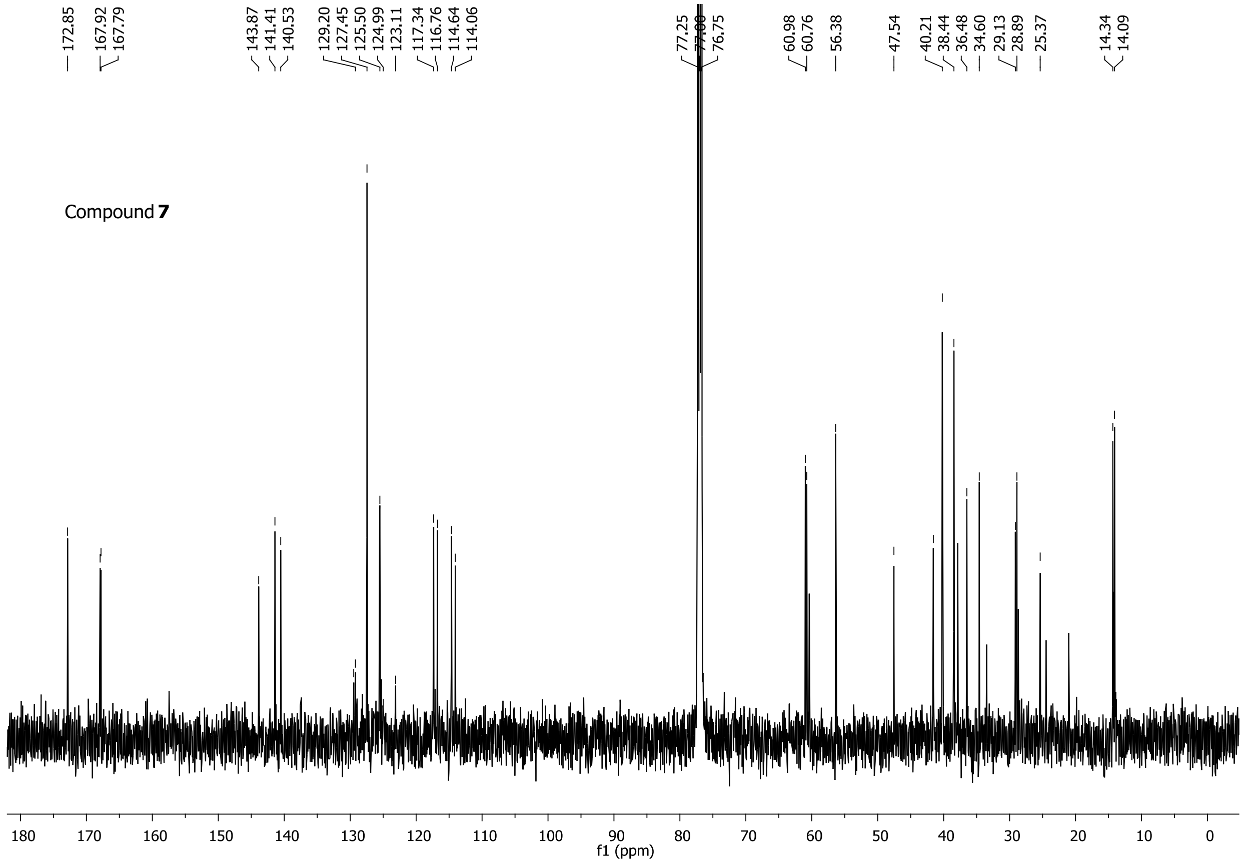
Compound 7
f1 (ppm)

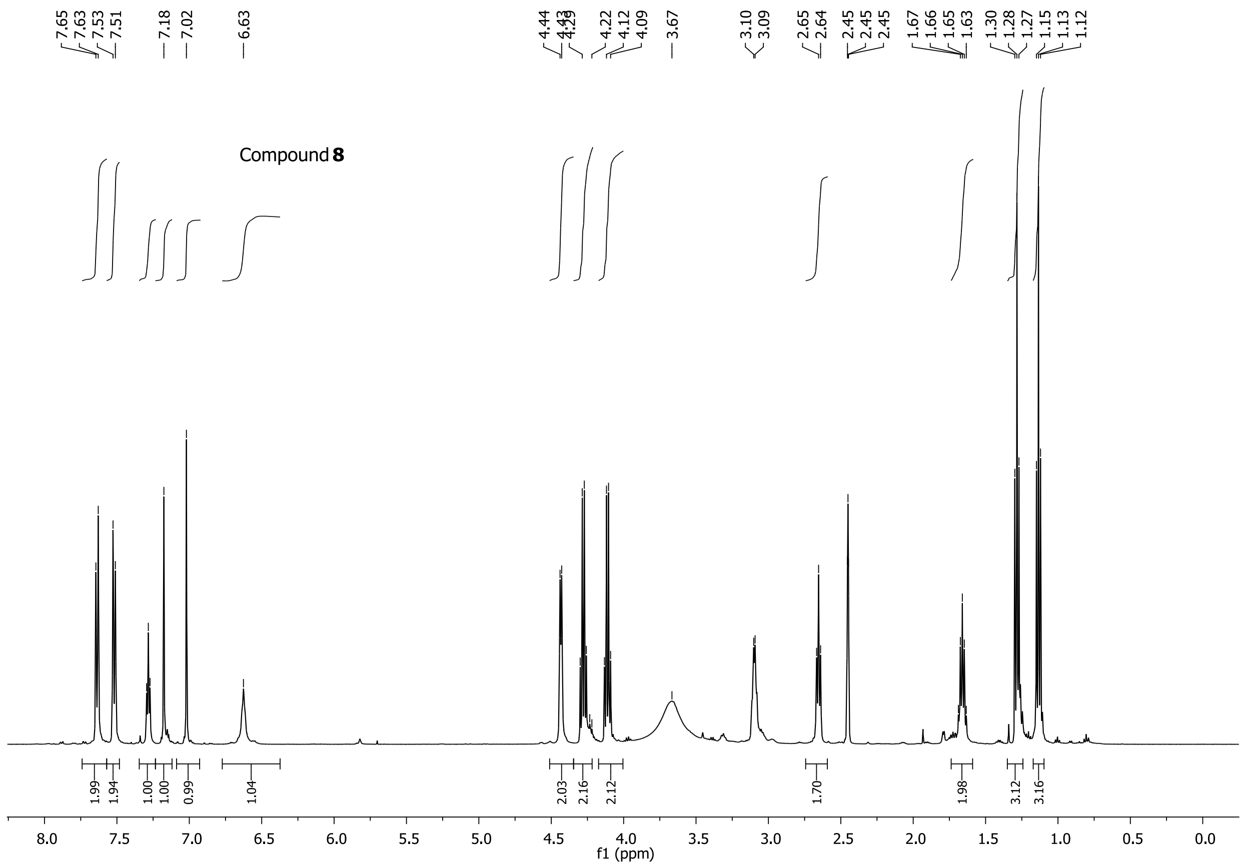

7.65
7.63
7.53
7.51
7.18
7.02
6.63
4.44
4.43
4.29
4.22
4.12
4.09
3.67
3.10
3.09
2.65
2.64
2.45
2.45
2.45
1.67
1.66
1.65
1.63
1.30
1.28
1.27
1.15
1.13
1.12
Compound **8**
1.99
1.94
1.00
1.00
0.99
1.04
2.03
2.16
2.12
1.70
1.98
3.12
3.16
8.0 7.5 7.0 6.5 6.0 5.5 5.0 4.5 4.0 3.5 3.0 2.5 2.0 1.5 1.0 0.5 0.0
f1 (ppm)

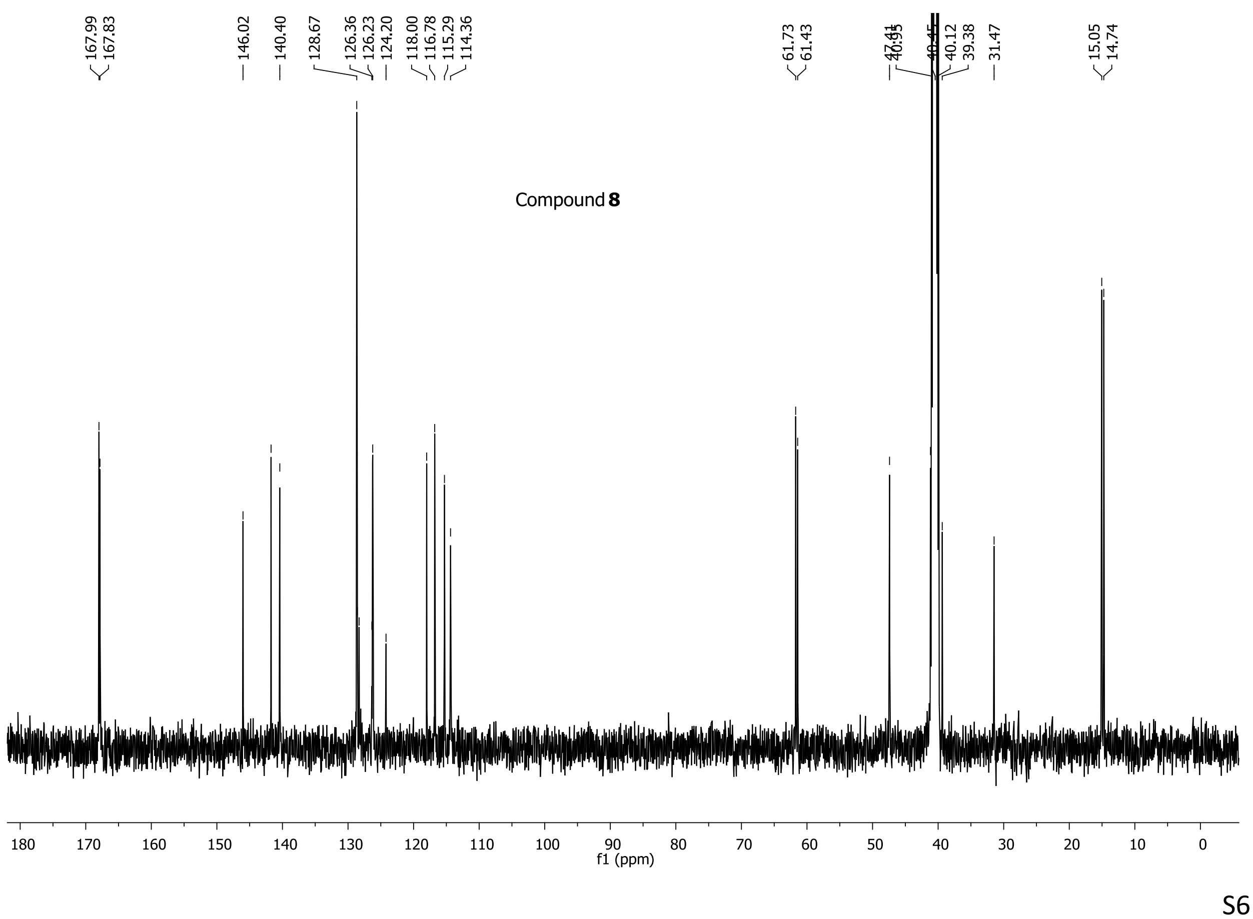

167.99
167.83
146.02
140.40
128.67
126.36
126.23
124.20
118.00
116.78
115.29
114.36
61.73
61.43
47.41
40.95
40.45
40.12
39.38
31.47
15.05
14.74
Compound **8**
180 170 160 150 140 130 120 110 100 90 80 70 60 50 40 30 20 10 0
f1 (ppm)
